# Effect on the tensile strength of human acellular dermis (Epiflex®) of in-vitro incubation simulating an open abdomen setting

**DOI:** 10.1186/1471-2482-14-7

**Published:** 2014-01-27

**Authors:** Mario Vitacolonna, Michael Mularczyk, Florian Herrle, Torsten J Schulze, Hans Haupt, Matthias Oechsner, Lothar R Pilz, Peter Hohenberger, Eric Dominic Rössner

**Affiliations:** 1 Division of Surgical Oncology and Thoracic Surgery, Department of Surgery, University Medical Centre Mannheim, Heidelberg University, Theodor Kutzer Ufer 1-3, 68167 Mannheim, Germany; 2 Center for structural Materials, State Material Testing Institute Darmstadt (MPA), Chair and Institute for Material Science (IfW), Technische Universität Darmstadt, Darmstadt, Germany; 3 German Red Cross Blood Service, Baden Württemberg-Hessen, Medical Faculty of Mannheim, Institute of Transfusion Medicine and Immunology, Heidelberg University, Mannheim, Germany; 4 Medical Faculty Mannheim, Heidelberg University, Heidelberg, Germany

**Keywords:** Acellular dermis, Open abdomen, Breaking strength, Biologicals

## Abstract

**Background:**

The use of human acellular dermis (hAD) to close open abdomen in the treatment process of severe peritonitis might be an alternative to standard care. This paper describes an investigation of the effects of fluids simulating an open abdomen environment on the biomechanical properties of Epiflex® a cell-free human dermis transplant.

**Methods:**

hAD was incubated in Ringers solution, blood, urine, upper gastrointestinal (upper GI) secretion and a peritonitis-like bacterial solution *in-vitro* for 3 weeks. At day 0, 7, 14 and 21 breaking strength was measured, tensile strength was calculated and standard fluorescence microscopy was performed.

**Results:**

hAD incubated in all five of the five fluids showed a decrease in mean breaking strength at day 21 when compared to day 0. However, upper GI secretion was the only incubation fluid that significantly reduced the mechanical strength of Epiflex after 21days of incubation when compared to incubation in Ringer’s solution.

**Conclusion:**

hAD may be a suitable material for closure of the open abdomen in the absence of upper GI leakage and pancreatic fistulae.

## Background

Acellular dermal products and transplants are starting to play a significant role in reconstructive surgery [1-3]. Human acellular dermis (hAD) may offer some advantages over xenogeneic material, such as reduced immunogenicity and increased safety with regard to potential prion infections [4]. Although the hAD Alloderm® has been extensively used outside of Europe and in particular in the USA [5-9], it is not approved for use in Germany where tissue transplants are required to meet the stringent safety requirements of the German drug law. Epiflex® is currently the only hAD approved for use as a medicinal product in Europe [4].

Dermis is rich in collagen of various subtypes [10] and its biomechanical strength is principally a function of the density and degree of hydration [11] and crosslinking [12] of the collagen fibers. These factors will also influence the extent to which a hAD retains its mechanical strength when incubated in aggressive fluids akin to those present in an infected open abdomen.

An open abdomen is defined as an abdominal wall fascial defect persisting after laparotomy. This condition may be induced, or accepted in case of a planned second look, for prevention of abdominal compartment syndrome or during “damage control surgery”, or it may simply be impossible to close the abdomen due to loss of domain, extensive abdominal wall resection or insufficient fascial stability in the case of peritonitis [13]. Due to the success of commercial and non-commercial vacuum therapy regimes in open abdomen management, fascial closure rates of up to 100% in young damage control trauma patients can be achieved [13,14]. Closure rates in multimorbid septic abdominal populations are approximately only 30%. In cases where fascial closure fails or vacuum therapy is not available the abdomen is traditionally closed with a synthetic mesh [15]. Since the abdominal compartment is usually contaminated in such patients, a resorbable mesh (e.g. Vicryl) is used. These meshes resorb in the time taken for the abdominal defect to be filled by granulation tissue and a planned ventral hernia is developing. This hernia will then be repaired after 6–12 months, when secondary wound healing finished and an aseptic condition is achieved, with non-resorbable, synthetic meshes and/or component separation techniques [16]. Application of a biological mesh such as hAD in the initial phase could be a novel approach in such patients. To function in this setting, a hAD must be sufficiently structurally resistant to the hostile environment of an open abdomen containing blood, urine or stool from fistulas and typical bacteria found in peritonitis patients. If such a treatment regime could obviate planned ventral hernias, this could reduce morbidity, and the requirement for revisions. Manufactures of these biological meshes are heavily advertising these for the closure of a septic open abdomen, claiming their biostability without a proper proof. The intention was the proof of principle and to identify conditions not suitable for a repair with acellular dermis.

The present study focuses on an *in-vitro* examination of the effect of incubation in Ringer’s solution (physiological solution serving as a control group), urine, blood, a bacteria mixture and upper gastrointestinal (upper GI) secretion on the mechanical strength of hAD.

## Methods

All of our research was carried out in compliance to the Helsinki declaration. The blood donation was approved by the local ethics committee ( Ethic Approval 87/04 of the Ethik Kommission II der Medizinischen Fakultät Mannheim).

### Human acellular dermis

Epiflex® (German Institute for Cell and Tissue Replacement, Berlin, Germany) was used as hAD. The transplant material used in the study originated from five screened and consenting human cadaveric donors. The mechanical processing, decellularization, sterilization and preservation methods [4] and collagen content [10] are described in detail elsewhere. All samples were derived from same body region. Each donor was randomized into one of the five groups (control, blood, urine, upper GI secretion and bacterial solution).

### Incubation fluids and culture conditions

The hAD were incubated in Ringer’s solution, in 1 of 3 different human body fluids; whole blood, duodenal secretion, urine or in a bacteria mixture consisting of enterococcus faecalis (gram+; streptococci), staphylococcus aureus (gram+; staphylococci), e.coli (gram-; enterobacteriaceae) and pseudomonas aeruginosa (gram-; nonfermenter). The bacterial strains were provided by the Institute for Medical Microbiology and Hygiene, (University Medical Center Mannheim) and added at a concentration of 1x10^5^ml^-1^ in Dulbecco’s Modified Eagle Medium (DMEM) with high glucose (4.5 g/l) and no additives (PAA, Germany), aliquoted and frozen at −80°C (mixed 1:1 with glycerol) for later use. Upper gastro intestinal secretion was collected from consenting patients during upper gastrointestinal endoscopy procedures. In all cases it was necessary to evacuate the upper GI in order to examine the intestinal mucosa. Upper GI pathology could be ruled out in all patients and none had been taking anti-acid medication. The secretions were pooled into volumes of 5 litres, aliquoted into 15 ml tubes and stored at −80°C. Urine was collected daily from healthy voluntary donors.

Blood was retrieved from the Institute of Transfusion Medicine and Immunology, German Red Cross Blood Service. Centrifugation of whole blood separates the samples into erythrocyte concentrate, plasma and a thrombocyte and leucocyte rich buffy coat. The latter are further used to create pooled thrombocyte concentrates. Due to use of mainly - antihypertensive - medication, the buffy coats and plasma were not permitted for use in patients. To simulate cellular blood composition, especially of leucocytes that are mainly regarded responsible for digestion of foreign bodies, the blood used in these experiments was composed of both buffy coat and plasma of volunteer blood donors.

### Culture conditions

The transplant samples were rehydrated in Ringer´s solution for 30 minutes at room temperature and then incubated at 37°C in 10 ml of one of the test fluids. The test fluid completely covered the hAD sample. Nine samples were incubated in each of the fluids. The incubation fluids were replaced on a daily basis. With the exception of the bacteria mixture, the dishes were supplemented with a 1:100 dilution of a Penicillin/Streptomycin mixture (PAA, Germany) to give a final concentration of 100 U/ml Penicillin and 0,1 mg/ml Strepavidin. Samples were incubated for 0 (briefly rinsed), 7, 14 and 21 days.

### Measurement of mechanical properties

**Figure 1:**
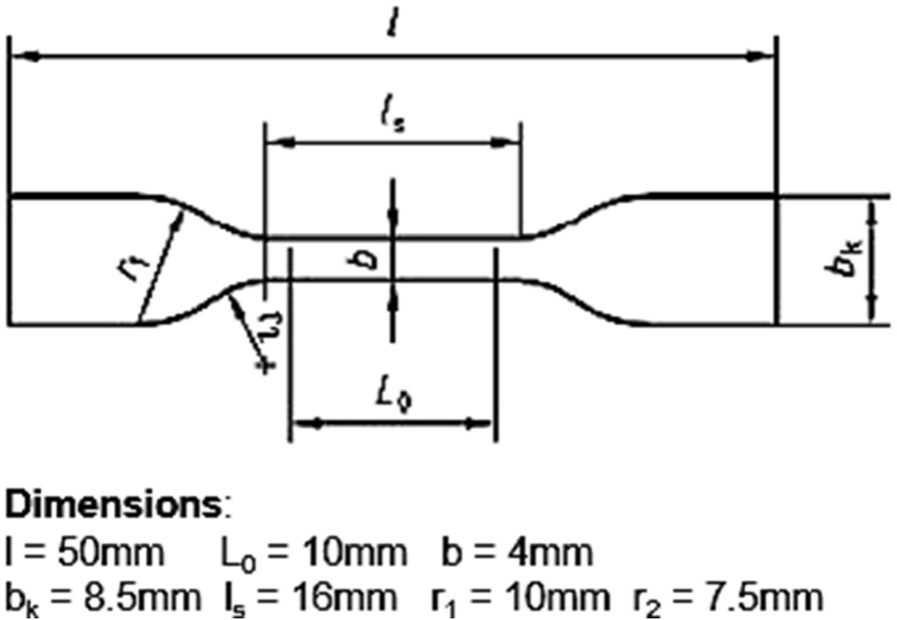
Dimensions of mechanical test specimens (ISO compliant).

**Figure 2:**
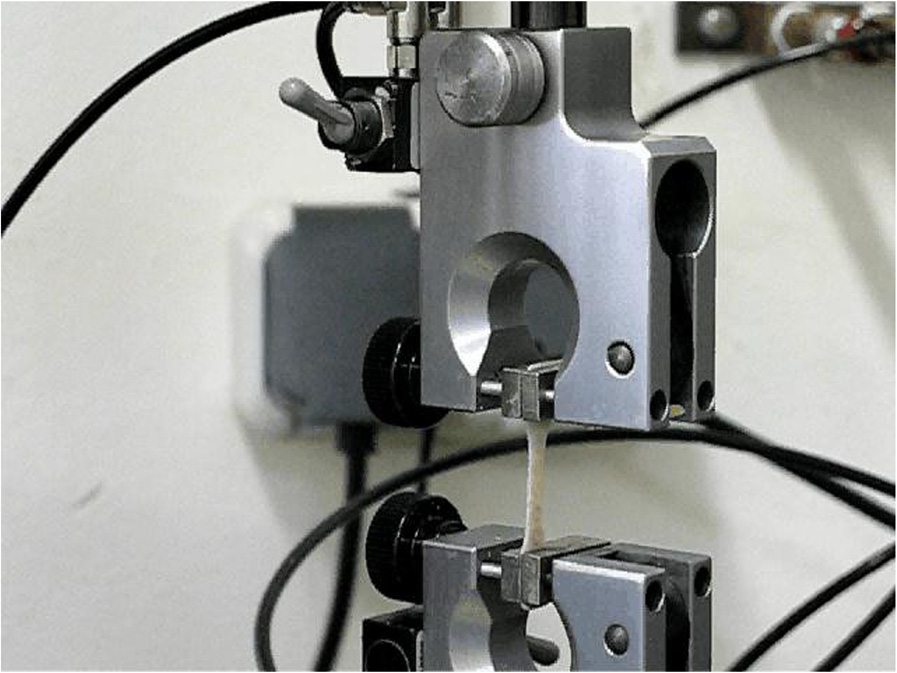
Sample clamped in the tensile testing machine.

Samples for mechanical testing were punched out of the transplants with a die cutter (Figure 1). The cut samples were submersed in Ringer´s solution for about 30 minutes prior to testing. The thickness of the mechanical test samples was measured at 5 points using a digital micrometer and the mean was calculated (JD200, Kaefer, Germany). The samples were evaluated in a tensile testing apparatus (H10KM, Richard Hess MBV GmbH, Sonsbeck, Germany) for ultimate load-at-failure according to EN ISO 527 (Figure 2). The test was carried out with a 100 N load cell at a constant strain rate of 50 mm/min.

### Histology

At the end of the respective incubation period, transplant samples were fixed in 10% formalin for 24 hours, sliced into 3 equal parts, embedded in paraffin and sectioned (7 μm) with a microtome (Microm, Germany) for a complete edge-to-edge cross- sectional view. Samples were mounted onto glass slides, dried overnight at 37°C, de- paraffinized with 3 washes of 5 minutes in a xylene bath followed by 3 washes of 2 minutes each through a dilution series of 100%, 96% and 80% ethanol. The autofluorescence of the samples was observed with a 488 nm excitation filter and a 514 nm emission filter in a fluorescence microscope (Zeiss, Germany) and stitched with ICE (Image Composite Editor, V1.4.4, Microsoft, USA).

### Statistics

For data collection and handling Excel 2010 was used (Microsoft, Redmond). Data was analyzed for normal distribution. A two-sided multiple Wilcoxon rank test with a correction according to Bonferroni- Holm for multiple testing was used. Tests were considered significant when α ≤ 0.05. For all statistics SAS (SAS Institute Inc., Carry, NC, USA) and StatXact 9 (Statcon, Witzenhausen, Germany) software was used. For the Koziol test the statistic system ADAM version 2.54 (DKFZ, Heidelberg, Germany) was used. Statistical analyses were conducted by a statistician of the Medical Faculty Mannheim, Heidelberg University, Germany.

## Results

**Figure 3:**
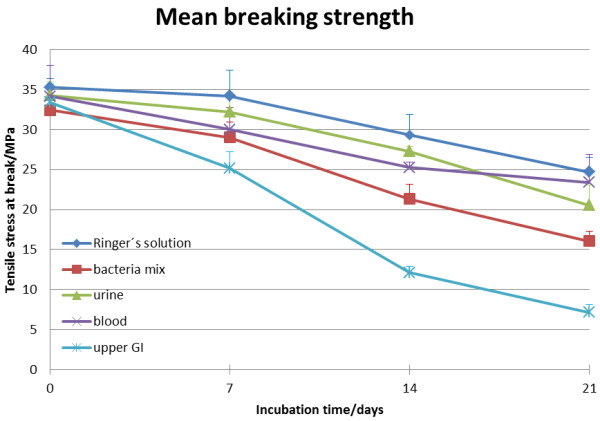
**Breaking strength of hAD.** The values are indicated as mean ± SEM.

Results are show in Figure 3. The breaking strength of hAD in Ringer’s Solution decreases over time. At day 21 the hAD has lost approximately 30% of its breaking strength. Although there is a continuous decrease of the mean breaking strength the differences between day 0 (35.28 ± 3.12 N/mm^2^) and day 7 (34.19 ± 3.50 N/mm^2^) and between day 14 (29.32 ± 2.67 N/mm^2^) and day 21 (24.69 ± 3.27 N/mm^2^) were not significant. In blood the breaking strength declines as well over time about 32%, although only the comparison between day 0 (34.14 ± 5.46 N/mm^2^) and 21 (23.38 ± 2.97 N/mm^2^) and between day 7 (30.0 ± 3.3 N/mm^2^) and day 21 were significant. A 40% decrease in breaking strength was measured for hAD incubated in urine for 21 days. The decrease was not significant between day 0 (34.3 ± 4.48 N/mm^2^) and day 7 (32.2 ± 4.215 N/mm^2^) and between day 7 and day 14 (27.29 ± 3.49 N/mm^2^). Incubation in a bacterial solution decreased the hAD breaking strength by 51% over a period of 21 days. Although the decrease from day 0 (32.42 ± 1.99 N/mm^2^) to 21 (16.06 ± 6.87 N/mm^2^) was significant the intervals from day 0 to 7 (29.01 ± 4.77 N/mm^2^), from day 7 to 14 (21.33 ± 2.54 N/mm^2^) and from day 14 to 21 were not significant. Breaking strength of hAD incubated in upper GI secretion showed the most distinct decrease of 78% over 21 days. All intervals showed a significant decrease in breaking strength. Mean breaking strength at day 0 was 33.42 ± 4.79 N/mm^2^, at day7: 25.19 ± 2.94 N/mm^2^, at day 14: 12.14 ± 2.045 N/mm^2^ and at day 21: 7.157 ± 2.84 N/mm^2^ (Table 1).

**Table 1:** Comparison of measured strength between paired time points within treatment groups ((+) significant difference) ((−) no significant difference)

	**Day 0 vs. 7**	**Day 7 vs. 14**	**Day 14 vs. 21**	**Day 0 vs. 21**
**Ringer’s solution**	-	+	-	+
**Blood**	-	-	-	+
**Urine**	-	-	+	+
**Bacteria**	-	-	-	+
**Upper GI secretion**	+	+	+	+

Comparing the breaking strengths of hAD in the different mediums at day 0 there were no significant differences. At day 7 the only significantly different treatment incubation in upper GI secretion. At day 14 both incubation in upper GI secretion and incubation in the bacteria solution resulted in mechanical strength being lower at the same time point than in the other 3 treatment groups. At this time point, hAD incubated in upper GI was significantly weaker than that incubated in the bacteria solution. At day 21 material incubated in upper GI secretion was again mechanically weaker than that in all other treatments. However, there were no longer any significantly differences amongst the samples incubated in Ringer’s solution, blood, urine and bacteria solution (Table 2).

**Table 2:** Comparison of measured strength at individual time points between treatment groups ((+) significant difference) ((−) no significant difference)

	**Day 0**	**Day 7**	**Day 14**	**Day 21**
**Ringer’s solution vs. blood**	-	-	-	-
**Ringer’s solution vs. urine**	-	-	-	-
**Ringer’s solution vs. bacteria**	-	-	+	-
**Ringer’s solution vs. GI secretion**	-	+	+	+

In the non-parametric analysis of the curves over time according to Koziol et al. the curve of the hAD incubated upper GI secretion is significantly (p = 0.0001) lower than the other curves.

**Figure 4:**
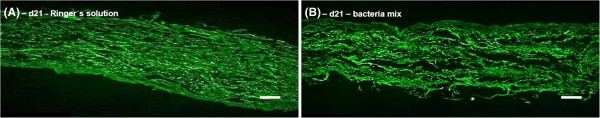
**Representatice fluorescence photomicrographs of hAD specimens incubated in (A) Ringer´s solution at day 21 and (B) in bacteria mix (collagen autofluorescence excitation 488 nm, emission 514 nm).** The figure shows a qualitative comparison of the matrix disaggregation incubated in control solution and bacteria mix. It can be seen, that matrix disaggregation in the bacteria group was more pronounced than in Ringer´s solution, presumably due to enzymatic cleavage (e.g. collagenases). Scale bars equal 50 μm.

The microscopy analysis of the hAD specimens showed disaggregation of the collagen fibers in all groups over time. In the bacterial and upper GI secretion group, disaggregation seems to occur more rapidly and more distinct when compared over time (Figure 4).

## Discussion

Since open abdomen is a therapeutic option in the treatment of traumatized abdomen or severe peritonitis there is a need for minimizing sequelae [17]. The well-established method of closure with a synthetic resorbable mesh prevents evisceration and chronic foreign body infection, although complication such as massive adhesions and small bowel fistulae are well known [18,19]. The need for major surgery to reconstruct the abdominal wall and for programmed ventral hernia raises the possibility of further morbidity, increased costs and mortality [20]. With introduction of intra-abdominal negative pressure dressings, delayed primary closure became an option in a high percentage of young trauma patients [21]. Delayed primary closure might be the best option in such patients, but in a majority of older patients with severe peritonitis due to septic focuses this is not feasible [22]. Closure with a tissue transplant could be helpful if adequate mechanical stability could be retained in an open abdomen environment.

Hollinsky and co-workers [23] measured the tensile strength of healthy human abdominal wall using specimens excised from fresh cadaver tissue. They were able to show that the linea alba fails in longitudinal and transversal direction at 39 N. This was calculated to be equivalent to a tensile strength of 10 N/mm^2^ and this may be regarded as the maximum strength required in a healthy human under extreme loads. The mean tensile strength of the transplants in the control group was 35 N/mm^2^ at day 0. Even 25 N/mm^2^, which was the mean strength after 21 days of incubation should be sufficient for Epiflex® to be able to withstand the maximum anticipated force, more so since the *in-vitro* test disregards adhesion and integration phenomena.

The breaking strength of hAD samples incubated in blood was not significantly different to that of transplants incubated in Ringer’s solution. Epiflex® should therefore be strong enough to reconstruct ventral hernias in an uninfected situation.

Epiflex® incubated in urine had similar properties. It should therefore be suitable for ventral reconstruction in the presence of an urinoma or urinary tract leakage.

Incubation in a bacteria solution resulted in no significant loss of strength at day 0 and 7 when compared to incubation in Ringer’s solution, but there was a significant difference at day 14. At day 21 there was no longer a significant difference. Typical intestinal flora may be capable of reducing the mechanical strength of Epiflex® within the time frame under investigation to a greater extent than blood or urine, although the residual mechanical strength of 16 N/mm^2^ still exceeds the calculated requirement.

The study has limitations with regard to the composition of the bacteria solution. Although the solution represents a common mixture of bacteria found in peritonitic patients, different bacteria mixtures could well have significantly different effects on hAD, since different bacteria strains and species excrete different concentrations of active agents such as collagenase.

The samples were incubated in high concentrations of bacteria that would only arise in an uncontrolled septic focus in the open abdomen. In such cases, an attempt at a primary closure would not normally be indicated. The combined effect of bacterial secretions and the inflammatory host response on the mechanical strength of candidate materials for abdominal wall closure cannot be simulated *in-vitro*.

Upper GI tract secretion had a powerful effect on the mechanical strength of Epiflex®. Loss of mechanical strength was continuous and when compared to the effects of the other incubation fluids, significantly increased at day 7, 14 and 21. The presence of an upper GI leakage, the closure of an open abdomen with Epiflex® might be compromised. At this stage of an open abdomen therapy, a definite closure is rarely indicated. It is unclear whether a pure pancreatic secretion from a pancreas fistula would have the same impact on the mechanical strength of Epiflex®.

The decrease in mechanical strength in the different liquids might be caused by various factors. Upper GI secretion is a mixture of gastric fluid, bile and pancreatic fluid and contains a heterogeneous mixture of digestive enzymes including proteases, lipases and amylases [24,25]. The upper GI secretion was frozen shortly after collection at −80°C to retard reduction of enzyme activity. Since the extracellular matrix consists of various proteins, glycoproteins and polysaccharides, enzymatic hydrolysis would seem to be a likely contributor to loss of tensile strength [26,27]. Bacteria also secrete hydrolytic enzymes such as collagenases, whereby the extent and the composition depends on species and strain. Furthermore, microbial organisms can modify the pH of the environment [28-30]. This may influence the degradation of bioresorbable materials [31-33]. It is known that superoxide ions from leukocytes and macrophages accelerate the degradation of absorbable materials [34]. The mechanism leading to loss of mechanical strength in Ringer's solution after 21 days is unclear. Temperature may affect biodegradation [35,36], however the incubation temperature in our study (37°C) seems unlikely to have exacerbated hydrolysis. Numerous studies demonstrated a significant loss in strength of biodegradable materials in aqueous solutions, presumably by cumulative low-level irreversible hydrolysis [33,36,37].

### Limitations of the studyy

In the clinical situation wound healing processes such adhesions to the transplant and integration, remodeling, vascularization, inflammation and scaring will have an effect on the mechanical strength of the transplant and the forming abdominal wall. This limits our findings especially after 21 days of incubation. Some of these effects are likely to be positive, although it cannot be ruled out that the remodeling process in itself results periods during healing during which mechanical strength is reduced, if resorption processes advance more rapidly than synthetic processes. The uniaxial tensile stresses applied to the transplants in our study do not ideally represent the stresses that occur in vivo. The latter are dynamic and multidirectional and can best be analyzed in a clinical setting.

## Conclusion

Epiflex® exhibits reduced mechanical strength after 3 weeks of incubation in Ringer’s solution, in body fluids (blood, urine, upper GI secretion) and in a bacteria solution. It seems unlikely that the loss of mechanical strength arising from incubation in Ringer’s solution, blood and urine would be clinically significant in the setting of a primary closure of an open abdomen. The loss of mechanical strength arising from incubation in a bacteria solution suggests that this might be clinically significant in an infected open abdomen situation, depending on the concentration, species and strain of the contaminating organisms. Incubation of Epiflex® in upper GI secretion caused a more pronounced loss of mechanical strength. Use of hAD for open abdomen closure in the presence of upper GI leakage or of a pancreatic fistula may be inappropriate. The authors intend to proceed with a phase I clinical study. The superiority of hAD in regards to the development of ventral hernia must be shown in a phase III study.

## Abbreviations

hAD: Human acellular dermis; Upper GI secretion (UGI): Upper gastro intestinal secretion; DMEM: Dulbecco’s Modified Eagle Medium; SEM: Standard error of the mean.

## Competing interests

The authors declare that they have no competing interests.

## Authors’ contributions

The study was conceived and designed by MV, ER and PH. The experiments were conducted and the results analyzed by MV, MM, FH, TJS, HH, MO and LRP. The manuscript was written by MV and ER. All authors read and approved the final manuscript.

## Pre-publication history

The pre-publication history for this paper can be accessed here:

http://www.biomedcentral.com/1471-2482/14/7/prepub

## References

[B1] Losee JE Smith DM Acellular dermal matrix in palatoplasty Aesthet Surg J 2011 31 7 Suppl 108S 115S 10.1177/1090820X11418216 21908830

[B2] Bengtson BP Baxter RA Emerging applications for acellular dermal matrices in mastopexy Clin Plast Surg 2012 39 2 159 166 10.1016/j.cps.2012.02.006 22482357

[B3] Taner T Cima RR Larson DW Dozois EJ Pemberton JH Wolff BG The use of human acellular dermal matrix for parastomal hernia repair in patients with inflammatory bowel disease: a novel technique to repair fascial defects Dis Colon Rectum 2009 52 2 349 354 10.1007/DCR.0b013e31819a3e69 19279435

[B4] Rossner E Smith MD Petschke B Schmidt K Vitacolonna M Syring C von Versen R Hohenberger P Epiflex((R)) a new decellularised human skin tissue transplant: manufacture and properties Cell Tissue Bank 2011 12 3 209 217 10.1007/s10561-010-9187-3 20574693

[B5] Scott BG Welsh FJ Pham HQ Carrick MM Liscum KR Granchi TS Wall MJ Jr Mattox KL Hirshberg A Early aggressive closure of the open abdomen J Trauma 2006 60 1 17 22 10.1097/01.ta.0000200861.96568.bb 16456431

[B6] Adetayo OA Salcedo SE Bahjri K Gupta SC A meta-analysis of outcomes using acellular dermal matrix in breast and abdominal wall reconstructions: event rates and risk factors predictive of complications Ann Plast Sur 2011 10.1097/SAP.0b013e31822afae5 22156884

[B7] Singh MK Rocca JP Rochon C Facciuto ME Sheiner PA Rodriguez-Davalos MI Open abdomen management with human acellular dermal matrix in liver transplant recipients Transplant Proceed 2008 40 10 3541 3544 10.1016/j.transproceed.2008.06.105 19100433

[B8] de Moya MA Dunham M Inaba K Bahouth H Alam HB Sultan B Namias N Long-term outcome of acellular dermal matrix when used for large traumatic open abdomen J Trauma 2008 65 2 349 353 10.1097/TA.0b013e31817fb782 18695470

[B9] Diaz JJ Jr Conquest AM Ferzoco SJ Vargo D Miller P Wu YC Donahue R Multi-institutional experience using human acellular dermal matrix for ventral hernia repair in a compromised surgical field Archiv Surgery 2009 144 3 209 215 10.1001/archsurg.2009.12 19289658

[B10] Roessner ED Vitacolonna M Hohenberger P Confocal laser scanning microscopy evaluation of an acellular dermis tissue transplant (Epiflex(R)) PloS One 2012 7 10 e45991 10.1371/journal.pone.0045991 23056225 PMC3462806

[B11] Ateshian GA Wang H Rolling resistance of articular cartilage due to interstitial fluid flow Proc Inst Mech Eng H 1997 211 5 419 424 10.1243/0954411971534548 9427837

[B12] Buehler MJ Nanomechanics of collagen fibrils under varying cross-link densities: atomistic and continuum studies J Mech Behav Biomed Mater 2008 1 1 59 67 10.1016/j.jmbbm.2007.04.001 19627772

[B13] Vargo D Richardson JD Campbell A Chang M Fabian T Franz M Kaplan M Moore F Reed RL Scott B Management of the open abdomen: from initial operation to definitive closure Am Surg 2009 75 11 S1 S22 19998714

[B14] Van Hensbroek Boele P Wind J Dijkgraaf MG Busch OR Carel Goslings J Temporary closure of the open abdomen: a systematic review on delayed primary fascial closure in patients with an open abdomen World J Surg 2009 33 2 199 207 10.1007/s00268-008-9867-3 19089494 PMC3259401

[B15] Regner JL Kobayashi L Coimbra R Surgical strategies for management of the open abdomen World J Surg 2012 36 3 497 510 10.1007/s00268-011-1203-7 21847684

[B16] Jernigan TW Fabian TC Croce MA Moore N Pritchard FE Minard G Bee TK Staged management of giant abdominal wall defects: acute and long-term results Annal Surg 2003 238 3 349 355 discussion 355–347 10.1097/01.sla.0000086544.42647.84 PMC1422713 14501501

[B17] Demetriades D Total management of the open abdomen Inter Wound J 2012 9 Suppl 1 17 24 10.1111/j.1742-481X.2012.01018.x PMC7950339 22727136

[B18] Bee TK Croce MA Magnotti LJ Zarzaur BL Maish GO 3rd Minard G Schroeppel TJ Fabian TC Temporary abdominal closure techniques: a prospective randomized trial comparing polyglactin 910 mesh and vacuum-assisted closure J Trauma 2008 65 2 337 342 discussion 342–334 10.1097/TA.0b013e31817fa451 18695468

[B19] Prichayudh S Sriussadaporn S Samorn P Pak-Art R Kritayakirana K Capin A Management of open abdomen with an absorbable mesh closure Surgery Today 2011 41 1 72 78 10.1007/s00595-009-4202-7 21191694

[B20] DeMaria EJ Moss JM Sugerman HJ Laparoscopic intraperitoneal polytetrafluoroethylene (PTFE) prosthetic patch repair of ventral hernia. Prospective comparison to open prefascial polypropylene mesh repair Surg Endos 2000 14 4 326 329 10.1007/s004640020013 10790548

[B21] Wondberg D Larusson HJ Metzger U Platz A Zingg U Treatment of the open abdomen with the commercially available vacuum-assisted closure system in patients with abdominal sepsis: low primary closure rate World J Surg 2008 32 12 2724 2729 10.1007/s00268-008-9762-y 18836762

[B22] Quyn AJ Johnston C Hall D Chambers A Arapova N Ogston S Amin AI The open abdomen and temporary abdominal closure systems - historical evolution and systematic review Col Dis Offic J Ass Coloproctol Gr Br Irel 2012 14 8 e429 e438 10.1111/j.1463-1318.2012.03045.x 22487141

[B23] Hollinsky C Sandberg S Measurement of the tensile strength of the ventral abdominal wall in comparison with scar tissue Clin Biomech (Bristol, Avon) 2007 22 1 88 92 10.1016/j.clinbiomech.2006.06.002 16904247

[B24] Muftuoglu MA Ozkan E Saglam A Effect of human pancreatic juice and bile on the tensile strength of suture materials Am J Surg 2004 188 2 200 203 10.1016/j.amjsurg.2003.12.068 15249253

[B25] Kalantzi L Goumas K Kalioras V Abrahamsson B Dressman JB Reppas C Characterization of the human upper gastrointestinal contents under conditions simulating bioavailability/bioequivalence studies Pharmaceut Res 2006 23 1 165 176 10.1007/s11095-005-8476-1 16308672

[B26] Tian F Appert HE Howard JM The disintegration of absorbable suture materials on exposure to human digestive juices: an update Am Surg 1994 60 4 287 291 8129252

[B27] Sugimachi K Sufian S Weiss MJ Pavlides CA Matsumoto T Evaluation of absorbable suture materials in biliary tract surgery Inter Surg 1978 63 3 135 139 415997

[B28] Mailman ML The efficacy of bacterial collagenase for the digestion of gingival tissue collagen J Den Res 1979 58 4 1424 10.1177/00220345790580042201 219048

[B29] Maclennan JD Mandl I Howes EL Bacterial digestion of collagen J Clin Invest 1953 32 12 1317 1322 10.1172/JCI102860 13108999 PMC438477

[B30] Chung E McPherson N Grant A Tensile strength of absorbable suture materials: in vitro analysis of the effects of pH and bacteria J Surg Edu 2009 66 4 208 211 10.1016/j.jsurg.2009.06.007 19896625

[B31] Chu CC Moncrief G An in vitro evaluation of the stability of mechanical properties of surgical suture materials in various pH conditions Annal Surg 1983 198 2 223 228 10.1097/00000658-198308000-00019 PMC1353084 6870380

[B32] Chu CC A comparison of the effect of pH on the biodegradation of two synthetic absorbable sutures Annal Surg 1982 195 1 55 59 10.1097/00000658-198201001-00009 PMC1352404 6275809

[B33] Chu CC The in-vitro degradation of poly(glycolic acid) sutures–effect of pH J Biomed Mat Res 1981 15 6 795 804 10.1002/jbm.820150604 6273445

[B34] Lee KH Chu CC The role of superoxide ions in the degradation of synthetic absorbable sutures J Biomed Mat Res 2000 49 1 25 35 10.1002/(SICI)1097-4636(200001)49:1<25::AID-JBM4>3.0.CO;2-I 10559743

[B35] Tomihata K Suzuki M Ikada Y The pH dependence of monofilament sutures on hydrolytic degradation J Biomed Mat Res 2001 58 5 511 518 10.1002/jbm.1048 11505425

[B36] Freudenberg S Rewerk S Kaess M Weiss C Dorn-Beinecke A Post S Biodegradation of absorbable sutures in body fluids and pH buffers Europ Res Euro Chirurg Fors Rech Chirurg Europ 2004 36 6 376 385 10.1159/000081648 15591748

[B37] Cam D Hyon SH Ikada Y Degradation of high molecular weight poly(L-lactide) in alkaline medium Biomaterials 1995 16 11 833 843 10.1016/0142-9612(95)94144-A 8527598

